# Computational Mechanism of Methyl Levulinate Conversion to γ-Valerolactone on UiO-66 Metal Organic Frameworks

**DOI:** 10.1021/acssuschemeng.1c08021

**Published:** 2022-03-04

**Authors:** Manuel A. Ortuño, Marcos Rellán-Piñeiro, Rafael Luque

**Affiliations:** † Centro Singular de Investigación en Química Biolóxica e Materiais Moleculares (CIQUS), Universidade de Santiago de Compostela, 15782 Santiago de Compostela, Spain; ‡ Institute of Chemical Research of Catalonia, ICIQ, and the Barcelona Institute of Science and Technology, BIST, Av. Països Catalans 16, 43007 Tarragona, Spain; § Departamento de Química Orgánica, Universidad de Córdoba, Campus de Rabanales, Edificio Marie Curie, E-14014 Córdoba, Spain; ∥ Peoples Friendship University of Russia (RUDN University), 6 Miklukho Maklaya str., 117198 Moscow, Russian Federation

**Keywords:** Density functional theory (DFT), Metal organic framework (MOF), Catalyst design, Biomass valorization, Catalytic transfer hydrogenation (CTH)

## Abstract

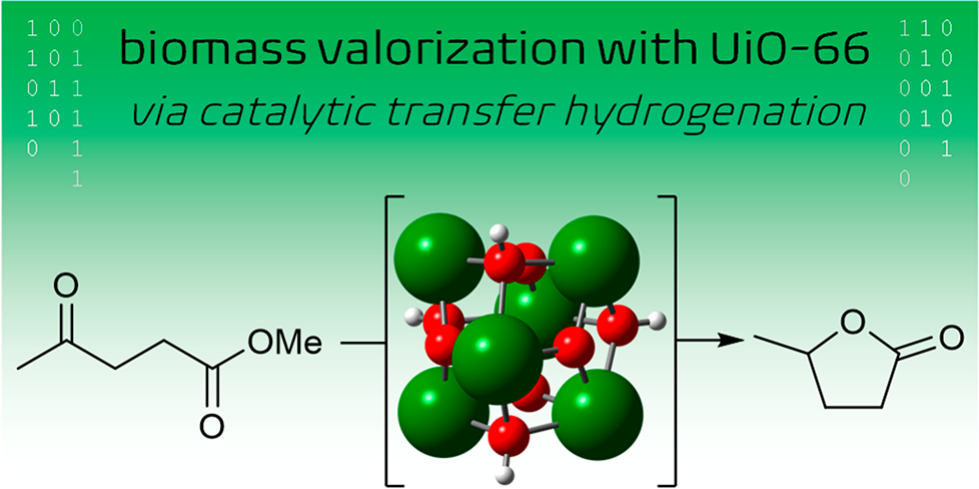

Metal–organic frameworks (MOFs) are gaining importance in the field of biomass conversion and valorization due to their porosity, well-defined active sites, and broad tunability. But for a proper catalyst design, we first need detailed insight of the system at the atomic level. Herein, we present the reaction mechanism of methyl levulinate to γ-valerolactone on Zr-based UiO-66 by means of periodic density functional theory (DFT). We demonstrate the role of Zr-based nodes in the catalytic transfer hydrogenation (CTH) and cyclization steps. From there, we perform a computational screening to reveal key catalyst modifications to improve the process, such as node doping and linker exchange.

## Abstract 2

Computational chemistry elucidates the mechanism to convert methyl levulinate into γ-valerolactone and guides the design of new metal organic framework catalysts.

## Introduction

The current scenario dominated by crude oil and natural gas is no longer feasible, and our society needs to devise new sustainable ways to fulfill the feedstock demand of an increasing world population. We must abandon the limited supply of fossil fuels and turn to renewable resources such as biomass. Indeed, the upgrading of readily available lignin and (hemi)cellulose to high-value products is already leading the way toward a sustainable economy.^1^ In that regard, several biobased molecules have been identified as promising building blocks.^2^ Among them, we here target the transformation of methyl levulinate (ML), readily available from lignocellulose, into γ-valerolactone (GVL), a platform molecule used as solvent, fuel, and feedstock for high-value chemicals.^3^

**Scheme 1:**
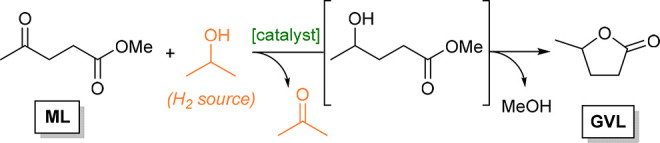
Conversion of Methyl Levulinate (ML) into γ-Valerolactone (GVL) via Catalytic Transfer Hydrogenation

This reaction is typically catalyzed in the heterogeneous phase and entails a direct hydrogenation via precious metals.^4^ Alternatively, amphoteric catalysts, such as those based on Zr, can promote a catalytic transfer hydrogenation (CTH) using alcohols as a hydrogen source (Scheme 1),^5,6^ thus avoiding the need of hydrogen gas and expensive metals. Zr-based oxides can indeed promote this process,^7,8^ but the variety of active sites on the catalyst surface may become detrimental due to undesired side reactions.

**Figure 1:**
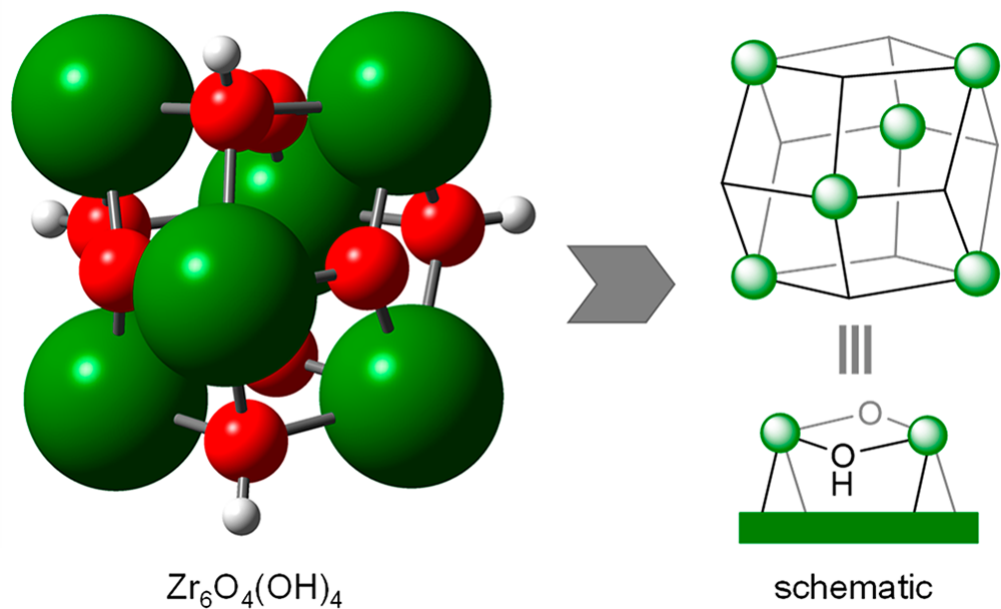
Representation of Zr-based nodes. Zr = dark green, O = red, H = white.

To better control the design of catalytic sites, we turn to metal–organic frameworks (MOFs), a family of porous materials that comprises inorganic nodes connected through organic linkers.^9^ Their high surface area, variable porosity, and well-defined coordination modes make them very valuable for general catalytic applications.^10^ This scope has been expanded to biomass conversion in recent years.^11,12^ Due to the thermal stability and catalytic properties of MOFs containing Zr_6_O_8_ nodes (Figure 1),^13^ they were tested in the valorization of alkyl levulinates to GVL via CTH. UiO-66 exhibited high activity and selectivity toward GVL in both batch^14^ and flow^15^ setups. Interestingly, while NH_2_- and COOH-functionalized linkers did not improve the catalytic performance,^14^ the presence of SO_3_H groups had a positive impact, presumably due to a cooperative effect between Lewis base nodes and Brønsted acid linkers.^16^ Other MOFs with similar Zr_6_O_8_ nodes (MOF-808,^14^ DUT-52,^17^ and ZrF^18^) can also catalyze this reaction with high conversion and moderate-to-good selectivity, as well as some Hf-based analogues.^19^ The improved catalytic activity obtained by MOF tuning is general and can be extrapolated to other processes, as recently demonstrated in UiO-66-catalyzed carbohydrate conversion.^20^

These promising results encourage further mechanistic understanding for catalyst optimization. Here is where computational chemistry comes into play to provide quantum mechanical insight at the atomic level of detail.^21^ Despite the many computational contributions on MOF catalysis,^22,23^ the mechanistic features of how these materials participate in biomass valorization are still scarce. Most of these studies employ finite-size clusters,^24^ which ignore the periodicity of the material and cannot account for confinement effects within the pores. To address this gap in knowledge, here we employ periodic density functional theory (DFT) to create a more realistic environment. We compute the reaction mechanism of ML to GVL at UiO-66 using isopropanol as a hydrogen source. We identify the key steps of the catalytic cycle and evaluate the impact of different catalyst modifications. Such mechanistic insight would guide the rational design of catalysts to develop more efficient and selective experimental systems.

## Computational Section

Calculations were performed at the periodic Density Functional Theory (DFT) level using the Vienna Ab-Initio Simulation Package (VASP).^25,26^ The PBE density functional^27^ was employed, and dispersion interactions were considered with Grimme’s D2 scheme.^28^ Core electrons were described by projector augmented wave (PAW) pseudopotentials,^29^ and valence electrons were represented by plane waves with a kinetic energy cutoff of 450 eV. A Hubbard correction^30^ of 4.5 eV was applied to Ce(4f) electrons as suggested in the literature.^31,32^ The simulation cell of UiO-66 (14.737 × 20.840 × 14.737 Å^3^, Figure S1) was taken from previous DFT calculations.^33^ The Brillouin zone was sampled at the Γ-point via the Monkhorst–Pack method.^34^ Transition state structures were located with the climbing image nudged elastic band^35^ and improved dimer^36^ algorithms. Minima and transition states were characterized by diagonalizing the numerical Hessian matrix (±0.015 Å displacements). Vibrational partition functions were computed using numerical frequencies at 513 K as in experiments,^15^ where only selected atoms were allowed to move.^37^

Open access^38^ to all inputs and outputs reported herein, including raw energies and geometries, is provided by the ioChem-BD platform^39^ in the following database.^40^

## Results and Discussion

The UiO-66 MOF is formed by Zr_6_O_4_(OH)_4_ nodes connected to 12 1,4-benzenedicarboxylate linkers.^41^ The pristine material does not have any open metal sites, but the presence of defects, i.e., missing linkers, is known to be responsible for catalytic activity.^42,43^ Thus, we first need to propose a feasible active site where the ML-to-GVL conversion may take place.

**Figure 2:**
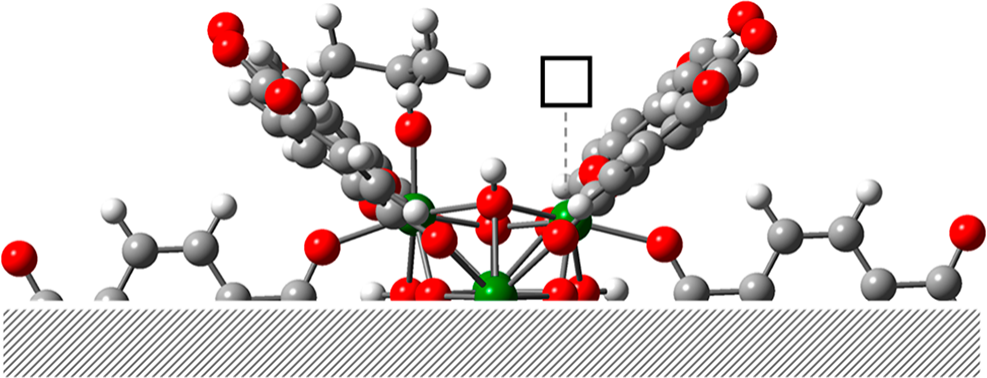
Computed structure **1** at defective UiO-66 MOF. The periodic structure was cropped for better visualization. The black square indicates a Zr vacant site. Zr = dark green, O = red, H = white.

The unit cell of UiO-66 contains four inorganic nodes.^41^ To save computational resources, we use a smaller cell containing two inorganic nodes.^33^ The removal of one dicarboxylate linker creates two node defects with four metal vacancies overall, which we then balance with OH/H_2_O groups.^44^ Under reaction conditions, we expect a displacement of H_2_O and exchange of [OH]^−^ by [^*i*^PrO]^−^, yielding the potential active species **1** (Figure 2). We will use this structure as a starting point for computing the reaction mechanism.

### Reaction Mechanism at Defective UiO-66

We start the mechanistic study from the previously discussed structure **1** which contains one ^*i*^PrO group and one Zr vacant site. We establish this stage as the zero of energies. The following values correspond to Gibbs energies computed at 513 K in eV. All species are denoted with numbers in bold, where transition states include the prefix **TS**.

**Figure 3:**
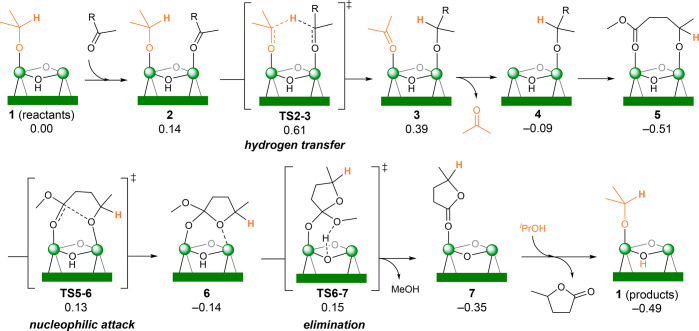
Reaction mechanism of ML to GVL at defective UiO-66 with relative Gibbs energies (in eV). R = (CH_2_)_2_CO_2_Me.

The proposed reaction mechanism is depicted in Figure 3 and entails catalytic transfer hydrogenation of ML followed by cyclization to GVL. First, species **1** binds ML through its carbonyl group forming **2** (0.14 eV). The hydrogen transfer from ^*i*^PrO to the activated ML occurs via **TS2–3** (0.61 eV) involving two Zr centers^45^ and yields intermediate **3** (0.39 eV). From there, acetone is released via **4** (−0.09 eV) and the alkoxide rearranges to form the bidentate species **5** (−0.51 eV), where the ester carbonyl group is also bound to Zr. An intramolecular nucleophilic attack via **TS5–6** (0.13 eV) generates the intermediate **6** (−0.14 eV). The subsequent elimination of MeOH is assisted by the μ_3_-OH group of the node.^46−48^ It takes place via **TS6–7** (0.15 eV) and results in the formation of bounded GVL in **7** (−0.35 eV). The nonassisted elimination via **TS6–8** (0.46 eV), which yields the Zr–OMe intermediate **8**, is less favored (Figure S2). Finally, an incoming ^*i*^PrOH reactant molecule releases the GVL product and regenerates catalyst **1**. The computed mechanism is in line with the experimental proposal, which involves two Zr atoms from the same node^14^ (rather than two Zr atoms from adjacent nodes^16^).

**Figure 4:**
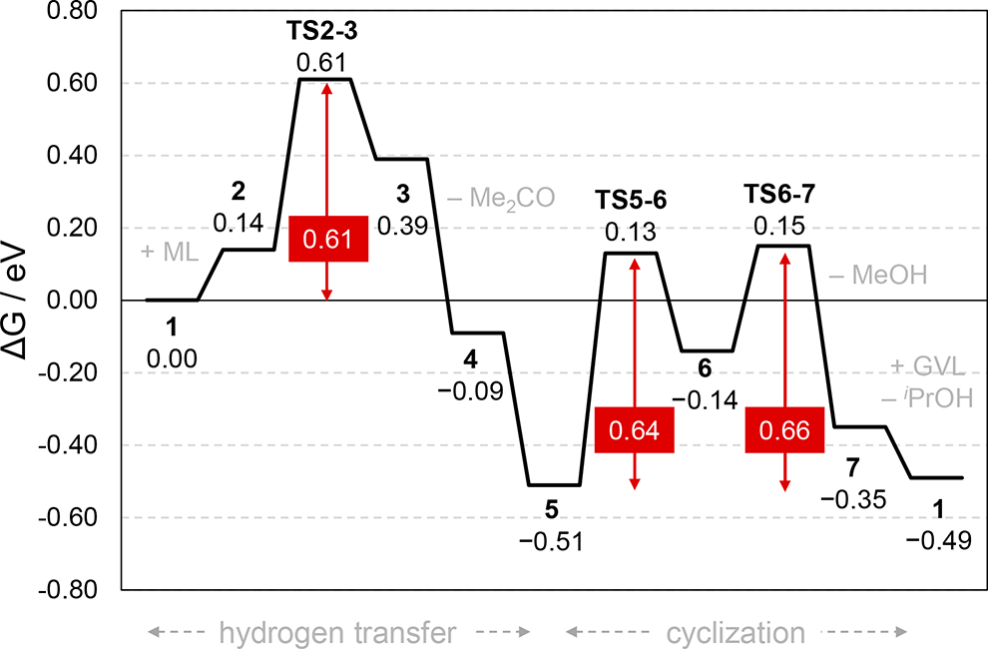
Gibbs energy reaction profile (in eV) at defective UiO-66 with relative barriers for each step.

The Gibbs energy profile of the reaction mechanism catalyzed by defective UiO-66 is displayed in Figure 4 (the electronic energy profile can be found in Figure S3). The coordination of ML to the catalyst **1** is slightly uphill by 0.14 eV, but this step is temperature-sensitive, and computed Gibbs energies typically over stabilize separated reactants due to entropic contributions. The hydrogen transfer **TS2–3** has a barrier of 0.61 eV above **1** and a relative barrier of 0.47 eV above **2**. Similar values were recently found in the CTH of furfural to furfuryl alcohol using finite-size cluster models of UiO-66^49^ and MOF-808.^50^ The release of acetone followed by the bidentate coordination of the substrate is quite favored, with **5** at 0.51 eV below **1**. Next, the cyclization takes place via nucleophilic attack and elimination of MeOH, where both **TS5–6** and **TS6–7** present similar barriers of 0.64 and 0.66 eV above **5**, respectively. The release of GVL and the addition of ^*i*^PrOH recover the catalyst **1** with global exoergic thermodynamics of 0.49 eV.

**Figure 5:**
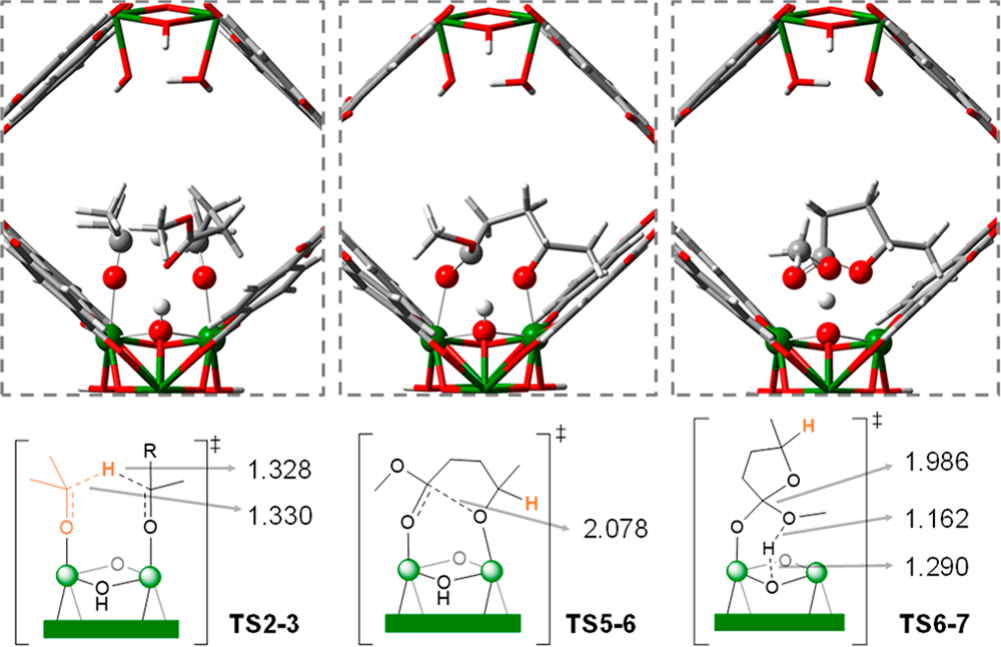
DFT-optimized TS structures. Relevant atoms are display in ball-and-stick format, the rest of them in tube format. Selected distances are shown in Å.

The structures of selected transition states confined within the MOF pore are displayed in Figure 5. **TS2–3** shows the hydrogen transfer; also, the ester group of ML forms a H bond with the μ_3_-OH group. **TS5–6** describes the intramolecular attack of the alkoxy to the carbonyl group. Finally, **TS6–7** represents the departure of the methoxy group and the concomitant abstraction of a proton from the μ_3_-OH group of the node. Further simulations at the PBE level with and without D2 dispersion corrections demonstrate the importance of such confinement effects (Figure S4), with **TS2–3** (hydrogen transfer) more affected than **TS5–6** and **TS6–7** (cyclization).

These simulations predict overall Gibbs energy barriers of ca. 0.65 eV for the UiO-66 catalyst. It demonstrates that the proposed mechanism is feasible under the reported experimental conditions, either in batch^14,16^ or flow reactors.^15^ They also predict that the cyclization process is likely rate-determining, which is in line with the detection of slight amounts of methyl 4-hydroxypentanoate.^15,16^ Due to the importance of the node in the mechanism, we next evaluate several MOF modifications that directly impact the Zr_6_O_8_ core: changing the nature of the metals (tuning the node) and changing the ligands bound to them (tuning the linker).

To facilitate future reading and comparison, from now on we will label all species of the original unmodified UiO-66 with the prefix **A** (e.g., **A-1**). We will use letters in bold for different modifications together with numbers in bold for intermediates and transition states. The correspondence between letters and modifications will be indicated in the following sections. The numeric notation does not change, and the correspondence between numbers and structures can be consulted in Figure 3.

### Tuning the Node

The previous reaction mechanism shows that Zr atoms efficiently act as Lewis acids for carbonyl groups. We then consider whether other M(IV) atoms (Hf, Ti, and Ce) can facilitate the reaction. Although such doping is not trivial from an experimental point of view, mixed-metal nodes^51,52^ and Ce-based nodes^53^ have been previously reported in the literature.

**Figure 6:**
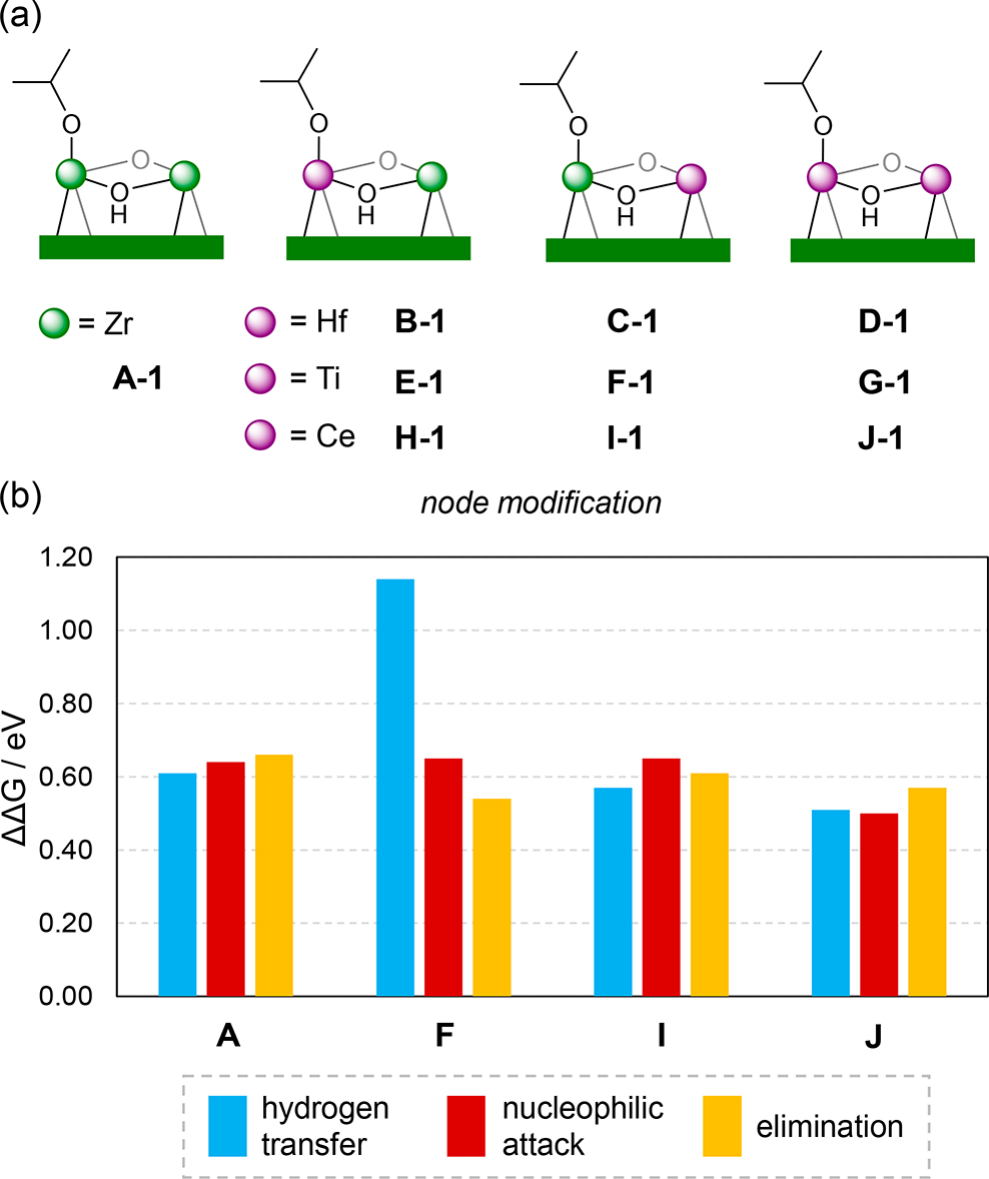
(a) Hf-, Ti-, and Ce-doped nodes and (b) relative Gibbs energy barriers (in eV) for selected models. Barriers are computed from the transition state to the previous most stable intermediate.

Considering the two Zr atoms involved in the process (**A**), we exchange them by Hf, Ti, and Ce (**B**–**J**) as shown in Figure 6a. We then compute energy barriers for each step: hydrogen transfer (from **1** to **TS2–3**), nucleophilic attack (from **5** to **TS5–6**), and elimination (from **5** to **TS6–7**). For the Hf derivatives **B**–**D**, the differences in electronic energy with respect to **A** are less than 0.05 eV (Figure S5), and we expect similar Gibbs energy profiles for both Zr- and Hf-based nodes. However, more changes are noted for Ti and Ce derivatives, and representative systems are shown in Figure 6b. For **F** with one Ti, the hydrogen transfer is significantly more demanding (**F-TS2–3** at 1.14 eV) due to a weak adsorption of ML (**F-2** at 0.50 eV), while the nucleophilic attack remains roughly the same and the elimination becomes easier (**F-TS6–7** at 0.38 eV, 0.54 eV above **F-5**). For **I** with one Ce, the hydrogen transfer and elimination steps are only marginally better, and the nucleophilic attack does not change. Interestingly, for **J**, which includes two Ce atoms, all barriers are reduced. The adsorption of ML is slightly stronger (**J-2** at −0.10 eV), and the hydrogen transfer barrier is lower (**J-TS2–3** at 0.41 eV, 0.51 eV above **J-2**). Likewise, the alkoxy intermediate is more stable and the cyclization process is overall faster.

To sum up, the presence of Ti can speed up the cyclization process at the expense of slowing down the hydrogen transfer. Ce-containing nodes provide a general decrease of barriers for all steps, in line with simulations on chemical warfare decomposition.^54^ With these results, we hypothesize that rare-earth-based MOFs^55^ could also exhibit similar or improved catalytic activity for biomass-related processes.

### Tuning the Linker

Not only nodes but also linkers can be fine-tuned in MOF catalysts. Therefore, we now consider changing the groups around the metals through linker modifications. Previous theoretical studies have pointed out that functionalizing the aromatic linkers has little effect on computed energy barriers^46,56^ and frequencies.^57^ We thus take a different approach and change the type of connectivity between the linker and the node.

**Figure 7:**
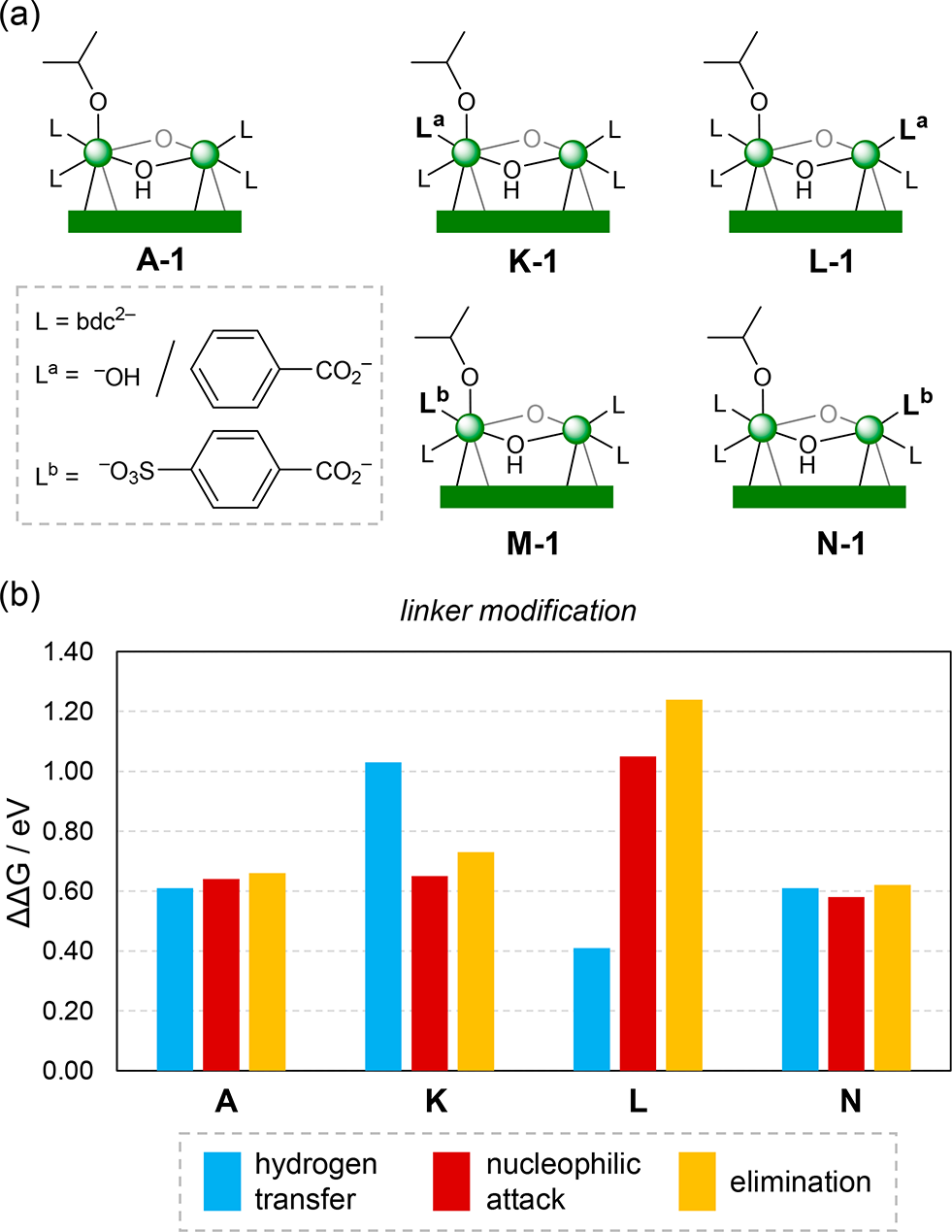
(a) Linker-modified nodes and (b) relative Gibbs energy barriers (in eV) for selected models. Barriers are computed from the transition state to the previous most stable intermediate.

As mentioned before, the unmodified MOF is labeled with the prefix **A**, where L is the original benzenedicarboylate (bdc). Considering the linkers bound to the participating Zr atoms, we exchange them by L^a^ and L^b^ as shown in Figure 7a. In L^a^, one bidentate carboxylate group is removed, and one monodentate hydroxo is bound to Zr (**K** and **L**), thus formally introducing a new vacant site at the node. In L^b^, one carboxylate group is substitute by one sulfonate group^58^ (**M** and **N**). The relative Gibbs energy barriers are summarized in Figure 7b. For **K**, the adsorption of ML does not change much (**K-2** at 0.25 eV) but the hydrogen transfer becomes more difficult (**K-TS2–3** at 1.03 eV). This is due to a stronger interaction between the ^*i*^PrO and the unsaturated Zr, which decreases the nucleophilicity of the former. On the other hand, for **L**, the adsorption of ML is enhanced at the unsaturated Zr (**L-2** at −0.39 eV), and the hydrogen transfer becomes faster (**L-TS2–3** at 0.41 eV above **L-2**). Although an electron deficient Zr is beneficial in the first step, it later binds to the alkoxy intermediate strongly (**L-5** at −1.57 eV), creating a thermodynamic sink that hinders the cyclization process. For **M** and **N**, both systems behave the same, and only **N** is discussed for simplicity. The sulfonate group in **N** does not introduce large changes, *cf*. the carboxylate group in **A**, and similar barriers are obtained in both cases.

Overall, these results indicate that the excess of vacancies at Zr atoms is counterproductive for activity, which may relate to the worse catalytic performance after losing organic linkers.^15^ They also suggest that the reaction is feasible with MOFs containing SO_3_-modified linkers. Although there is no strong improvement in terms of computed reaction barriers, these materials may present other advantages from a practical point of view, such as higher number of defects along the framework (i.e., more catalytic sites) while increasing the stability of the material, as reported recently.^58^

## Conclusions

In this work, we study the role of UiO-66 in the conversion of methyl levulinate to γ-valerolactone using isopropanol as a hydrogen source. By means of periodic DFT simulations, we propose a feasible reaction mechanism in agreement with experiments, which consists of transfer hydrogenation and cyclization (nucleophilic attack followed by elimination). We find similar Gibbs energy barriers for all steps, thus no unique rate-determining step can be ascribed. Notably, the μ_3_-OH group at the node actively participates in the reaction by forming H-bonds with reactants and assisting in the elimination process.

As for design, we explore several catalysts by systematically varying the metal atoms in the node as well as the connecting group between the linker and the node. Our computational approach provides a precise control of MOF changes that allows us to inspect the impact of each modification on each step of the mechanism. In this way, we demonstrate that Ti (a hard Lewis acid) improves the hydrogen transfer but is detrimental for the cyclization, while highly unsaturated Zr operates the other way around. We also find that Ce-based nodes decrease all three reaction barriers and exploring related MOFs containing rare-earth elements would be interesting. Finally, Zr nodes with SO_3_-modified linkers are competitive, *cf*. the parent material, and can improve the overall performance of the catalytic system.

## Supporting Information Available

Alternative mechanism, electronic energy profile, additional energy barriers (PDF)
