# Supplementary material for: Computational Mechanism of Methyl Levulinate Conversion to γ-Valerolactone on UiO-66 Metal Organic Frameworks
Source: ACS Sustain Chem Eng. 2022 Mar 4;10(11):3567–73. doi: 10.1021/acssuschemeng.1c08021 (PMC8942187; doi:10.1021/acssuschemeng.1c08021)
Supplement: Supplementary file 1 — sc1c08021_si_001.pdf [file sc1c08021_si_001.pdf]

SUPPORTING INFORMATION

COMPUTATIONAL MECHANISM OF METHYL LEVULINATE CONVERSION TO γ-VALEROLACTONE ON UiO-66 METAL ORGANIC FRAMEWORKS

Manuel A. Ortuño*[,a,b] Marcos Rellán-Piñeiro,[a] Rafael Luque[c,d]

[a] Centro Singular de Investigación en Química Biolóxica e Materiais Moleculares (CIQUS), Universidade de Santiago de Compostela, 15782 Santiago de Compostela, Spain

[b] Institute of Chemical Research of Catalonia, ICIQ, and the Barcelona Institute of Science and Technology, BIST, Av. Països Catalans 16, 43007 Tarragona, Spain

[c] Departamento de Química Orgánica, Universidad de Córdoba, Campus de Rabanales, Edificio Marie Curie, E-14014 Córdoba, Spain

[d] RUDN University, 6 Miklukho Maklaya str., 117198 Moscow, Russian Federation

* E-mail: manuelangel.ortuno@usc.es

Number of pages: 7

Number of figures: 5

Number of tables: 2

Table of Contents

1. Computed unit cell of UiO-66 .... S3

2. Alternative elimination pathway S3

3. Electronic energy reaction profiles S4

4. Confinement effects S5

5. Relative barriers for Hf-based UiO-66 MOFs S6

6. Summary of Gibbs energy barriers S6

7. Desorption energies S7

8. Computed energies and structures S7

1. Computed unit cell of UiO-66

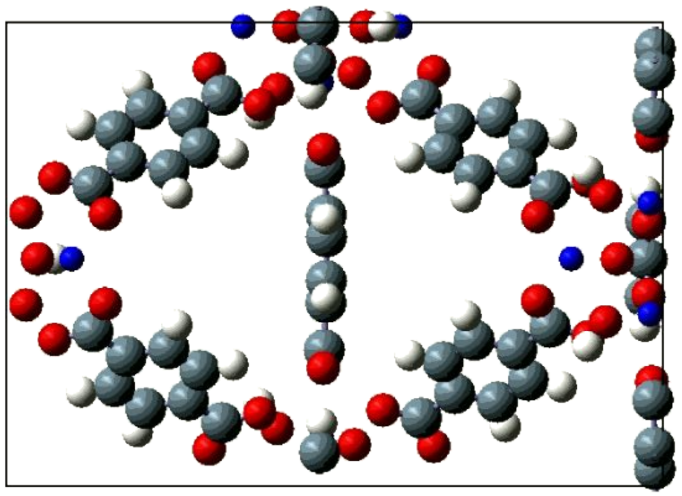

Figure S1. Representation of the periodic unit cell of UiO-66 used in these simulations.

2. Alternative elimination pathway

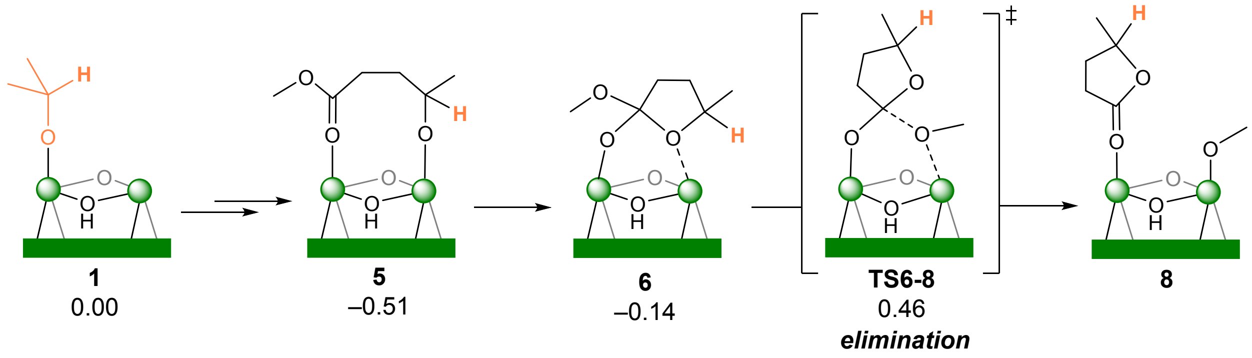

Figure S2. Alternative reaction pathway for the elimination with relative Gibbs energies (in eV).

3. Electronic energy reaction profiles

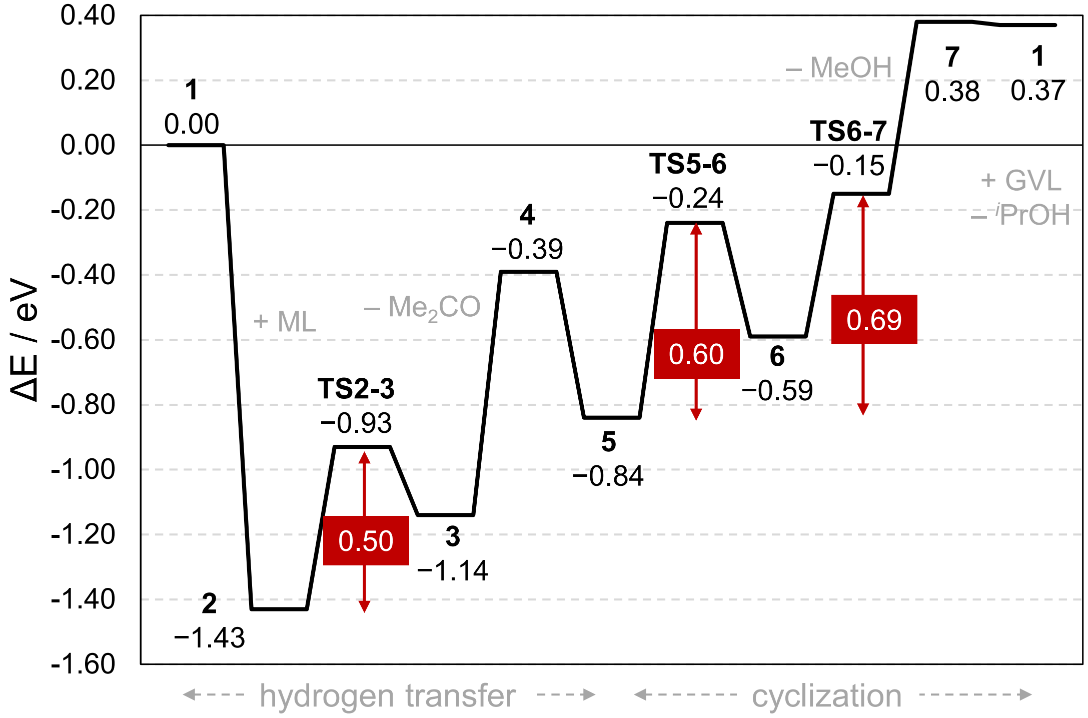

Figure S3. Electronic energy reaction profile (in eV) at defective UiO-66. Selected relative barriers are indicated to facilitate comparison with the Gibbs energy reaction profile.

4. Confinement effects

Figure S4 compares the electronic energy profiles computed with PBE and D2 dispersion (black line) and PBE without dispersion (purple line). The major difference appears in the hydrogen transfer step (0.8–1.0 eV), where methyl levulinate and iPrO are both bound to the node (**2**, **TS2-3**, and **3**). The PBE method does not account for dispersion interactions between substrate–substrate and substrate–MOF, thus the profile is shifted upwards. A similar trend is observed for the cyclization step (0.4–0.6 eV), but it is less marked since acetone has been released and only one bidentate ligand is found on the MOF node (**5**, **TS5-6**, **6**, **TS6-7**). Finally, there are only minor energy differences (0.1–0.2 eV) for species where the MOF only binds one monodentate ligand (**1**, **4**, **7**). Overall, the confinement effects driven by dispersion interactions are quite relevant and must not be ignored in the simulations.

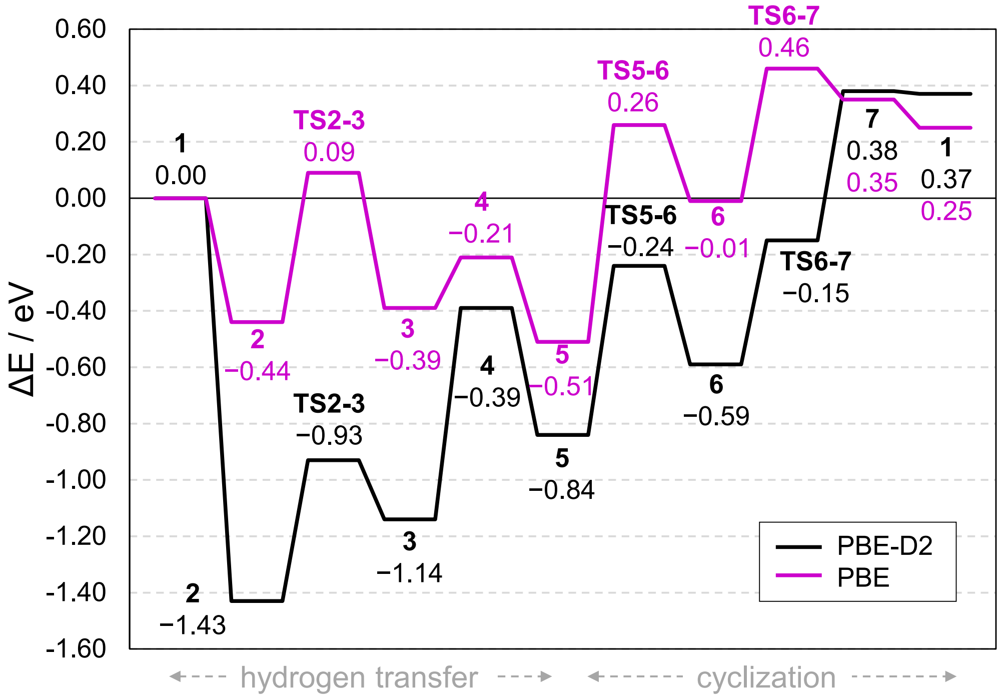

Figure S4. Electronic energy reaction profile (in eV) at defective UiO-66 at PBE-D2 (black line) and single-point PBE without dispersion (purple line).

5. Relative barriers for Hf-based UiO-66 MOFs

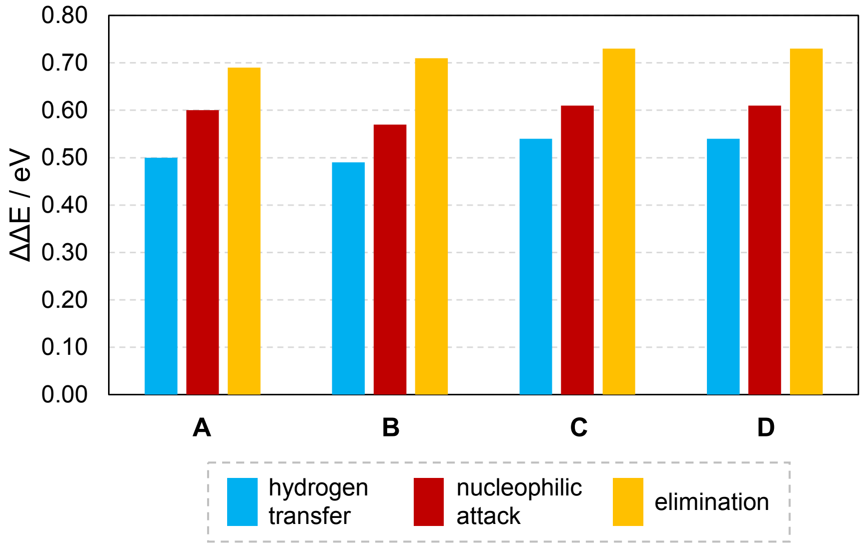

Figure S5. Relative electronic energy barriers for Zr_6O_8 node (**A**) and Hf-doped nodes (**B**–**D**).

6. Summary of Gibbs energy barriers

Table S1. List of overall Gibbs energy barriers in eV.

Step	*Hydrogen transfer*		*Nucleophilic attack*		*Elimination*	
Catalyst	species	ΔG	species	ΔG	species	ΔG
**A**	**TS2-3 – 1**	0.61	**TS5-6 – 5**	0.64	**TS6-7 – 5**	0.66
**F**	**TS2-3 – 1**	1.14	**TS5-6 – 5**	0.65	**TS6-7 – 5**	0.54
**I**	**TS2-3 – 1**	0.57	**TS5-6 – 5**	0.65	**TS6-7 – 5**	0.61
**J**	**TS2-3 – 2**	0.51	**TS5-6 – 5**	0.50	**TS6-7 – 5**	0.57
**K**	**TS2-3 – 1**	1.03	**TS5-6 – 5**	0.65	**TS6-7 – 5**	0.73
**L**	**TS2-3 – 2**	0.41	**TS5-6 – 5**	1.05	**TS6-7 – 5**	1.24
**N**	**TS2-3 – 1**	0.61	**TS5-6 – 5**	0.58	**TS6-7 – 5**	0.62

7. Desorption energies

Desorption energies (from **7** to **1**) for selected catalysts are listed below. In most cases the process is isoenergetic or slightly endothermic and can be easily surmounted under reaction conditions. The exception is **K**, where the process is exothermic due to the presence of additional defects in the node.

Table S2. List of overall Gibbs energy barriers in eV.

Step	*Desorption of GVL*
Catalyst	ΔG
**A**	−0.14
**F**	0.02
**I**	0.00
**J**	0.03
**K**	−0.76
**L**	0.19
**N**	0.14

8. Computed energies and structures

All computational raw data (inputs, outputs, energies, geometries) can be consulted free-of-charge in the open access repository ioChem-BD. See main text for the link to the database.
